# Structural design and performance study of primitive triply periodic minimal surfaces Ti6Al4V biomimetic scaffold

**DOI:** 10.1038/s41598-022-17066-6

**Published:** 2022-07-26

**Authors:** Yaru Qin, Qihui Wang, Chenglong Shi, Bing Liu, Shuqing Ma, Miao Zhang

**Affiliations:** 1 School of Chemistry and Chemical Engineering, Qinghai Minzu University, Xining, 810007 Qinghai China; 2 College of Chemical Engineering, North China University of Science and Technology, Tangshan, 063210 Hebei China

**Keywords:** Trauma, Composites, Metals and alloys, Computational methods, Predictive medicine, Preclinical research

## Abstract

This paper comprehensively evaluated the static mechanical compressive properties, permeability, and cell adhesion effect on the inner wall of the Primitive triply periodic minimal surface Ti6Al4V bionic scaffolds with different axial diameter ratios through numerical simulation and experiments. The results show that when the axial diameter ratio is 1:2, the elastic modulus of the scaffold is about 1.25 and the yield strength is about 1.36. The scaffold's longitudinal and transverse mechanical properties align with human bone tissue. Its permeability is also better than that of circular pores. The scaffold with an axial diameter ratio of 1:3 has the best permeability, ranging from 1.28e−8 to 1.60e−8 m^2^, which is more conducive to the adsorption of cells on the inner wall of the scaffold. These results show that the scaffold structure with an axial diameter ratio of not 1:1 has more advantages than the ordinary uniform scaffold structure with an axial diameter ratio of 1:1. This is of great significance to the optimal design of scaffold.

## Introduction

The repair of large bone defects has always been a difficult problem in clinics^1^. The biomimetic bone scaffold is considered to be the most promising way to solve this problem^1,2^. How to solve this problem, the key lies in the structure and material design of the bone scaffold. The real human bone tissue structure is a very complex pore structure, the pore size distribution is uneven, and the pore shape is also irregular, somewhat similar to elliptical pores^3^. Therefore, it is difficult to construct and process a structure that is completely consistent with real human bone tissue. In terms of materials, bone material is a kind of natural compound, which is mainly composed of all kinds of collagen and hydroxyapatite^4^. Of course, the similarity of structure and material alone can not meet the clinical requirements. The performance of the scaffold must match the real human bone tissue. The most important properties of human bone tissue are mechanical properties and permeability properties. The compressive strength of the scaffold must meet the load-bearing requirements because the scaffold should play a load-bearing role after being implanted into the human body. In addition, the scaffold must have sufficient ability to conduct fluid flow, and the pore structure of the scaffold should be suitable for cell adhesion and proliferation^5^.

In the early years, due to the lack of in-depth understanding and immature processing technology, the bone scaffold structure was often relatively simple, the most common was a variety of cube structures, and the material was mostly metal materials^6,7^. Which was not achieve good experimental and clinical results. In recent years, with the continuous development of the field of tissue engineering and the continuous improvement of medical 3D printing technology, the scaffold structure closer to the real human bone tissue has been designed and manufactured. Zhang et al. studied the diamond lattice pore structure and optimized the scaffold structure design by the finite element method^8^. Chen et al. studied some regular low gap structures and explored scaffold cell proliferation^9^. Montazeriana et al. studied the hexagonal and prismatic dodecahedron structures and explored the relationship between permeability and porosity^10^. Among them, the minimal surface structure was considered an excellent bone scaffold structure because of its large specific surface area and high porosity. Ataee et al. designed the commercial titanium gyroid structure and studied its mechanical compression response^11^. Yu et al. studied a variety of homogeneous minimal surface structures and studied the mechanical properties and permeability^12^. The research on materials was more and more extensive. Including a variety of metal materials, polymer materials, polymer materials, and ceramic materials^6,7,9,11,13–15^. Titanium alloys were widely used because of their good biocompatibility^16^.

Previous studies^4,6,7^ have shown that the pore structure of human bone tissue is irregular, the distribution is uneven, and the mechanical properties are different in all directions. Therefore, it is difficult to realize the anisotropy of mechanical properties through regular symmetrical pore structure.

In this study, considering that the pore structure of bone tissue is similar to ellipse, so we optimized the Primitive minimal surface with the different proportions of elliptical pore structure, and the Ti6Al4V material with excellent biological properties was selected. Through numerical simulation combined with experiments, we evaluated the transverse and longitudinal compressive strength and permeability of the designed scaffold structure and whether the permeability matches the real human bone tissue. The cell adhesion effects of different pore structures were evaluated to provide a reliable reference for the design of biomimetic bone scaffolds.

## Materials and methods

### Structure design method

**Figure 1:**
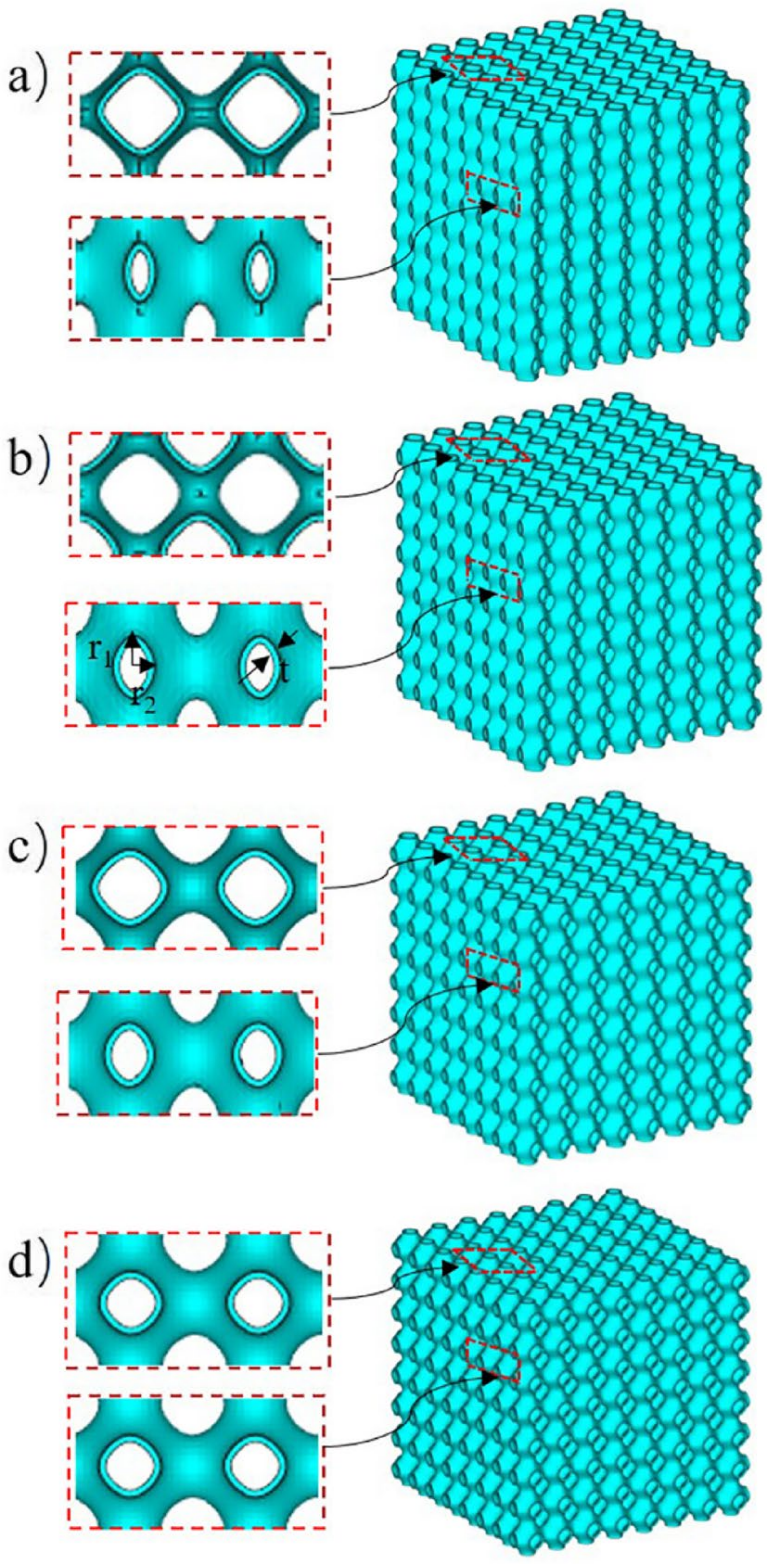
CAD diagram of four groups of scaffolds.

The scaffold models were generated by rhino software (Robert McNeel & Assoc, USA), as shown in Fig. 1. The unit size was 1 mm, the solid models were obtained by the wall offset 0.05 mm, and the surface structure was defined by the implicit function expression (1). Four groups of scaffold structures with different aspect ratios were designed, Fig. 1a–d, r1: r2 is 3:1, 2:1, 2:3, and 1:1 respectively. To facilitate the following description, the four groups of scaffolds were named and simplified according to the characteristics of their shaft-diameter ratio. They were referred to as P1-3 scaffold, P1-2 scaffold, P2-3 scaffold, and P1-1 scaffold respectively.

1 $${\varphi }_{D} (x,y,z) ={\lambda }_{1} {cos}\left(x\right)+{\lambda }_{2} {cos}\left(y\right)+{\lambda }_{3}{cos}\left(z\right)+\mu $$

where $\uplambda$ and $\upmu$ are constants.

### Simulation analysis of mechanical compression test

**Figure 2:**
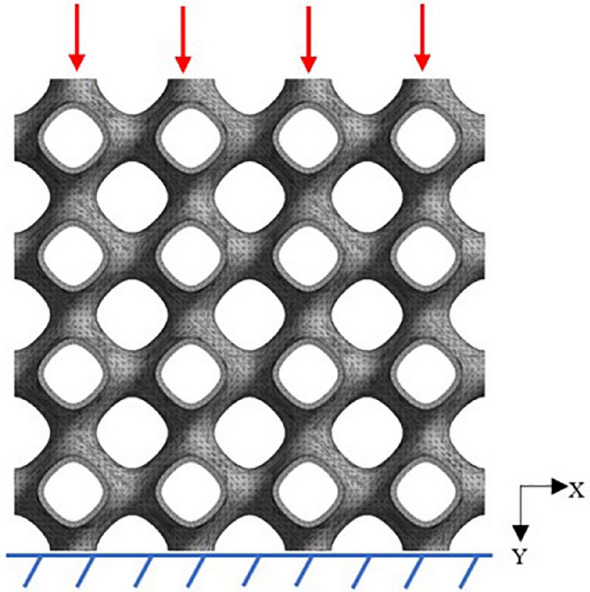
Finite element analysis model of porous scaffold.

Bone scaffold is an important load-bearing structure, so the compression characteristics are important. In this paper, the simulation process was completed in the software ABAQUS2016 (SIMULIA, America, https://www.onlinedown.net/soft/10033228.htm). The boundary conditions were shown in Fig. 2. The whole lower surface of the scaffold was fixed and restrained, and the upper surface is uniformly loaded with a displacement load of 0.096 mm along the Z-axis at a speed of 0.05 mm/min. The loading speed is consistent with the loading conditions of the micro-control electronic universal testing machine used in subsequent experiments. The transverse and longitudinal mechanical properties of each group of scaffolds were studied respectively. The loading conditions of the four groups of scaffolds were the same, and the static characteristics of each group of scaffolds were calculated and compared. In the simulation process, the bone scaffold material was Ti6Al4V, and the material parameters were shown in Table 1^17,18^.

**Table 1:** Properties of Ti6Al4V materials in finite element analysis.

Materials	Density (kg/m^3^)	Young’s modulus (GPa)	Yield strength (MPa)	Poisson’s ratio
Ti6Al4V	4430	113.8	970	0.32

### Preparation of model

**Figure 3:**
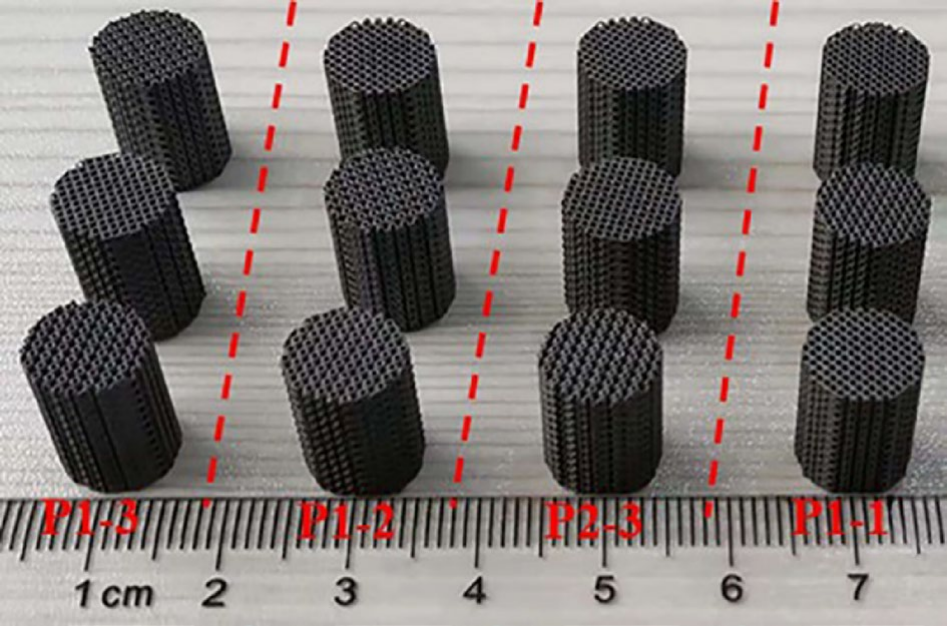
Four groups of Ti6Al4V samples manufactured by SLM.

Four groups of scaffold models were prepared by the SLM process. Ti6Al4V powder was purchased from Gaoke New Material Technology (Beijing) Co., LTD. The particle size is 12–50 μm. The 3D printing equipment used the SLM280 of Shanghai Kewei forming Technology Co., Ltd., which is equipped with a 500 W laser with a spot size of 60 μm. The scanning layer thickness and scanning speeds are 15 μm and 400 mm/s, respectively. Through the Boolean operation, the size of each sample was set to φ10 × 12. Four groups of representative scaffold models were shown in Fig. 3.

### Mechanical property test

The compression test of the scaffolds was carried out according to the International Standard of Metal Compression Test (ISO13314:2011). Four groups of samples were compressed at the compression speed of 0.05 mm/min by using a micro-controlled electronic universal testing machine. The stress–strain curves of each group of samples were drawn, and the elastic modulus and yield strength of each group of samples were obtained to evaluate the mechanical properties of each group of samples.

### Fluid dynamics simulation analysis

Permeability is one of the important characteristics of bone scaffold structure, which affects the effective transportation of oxygen and nutrients in the scaffold^19^. It is not conducive to the flow of fluid in the scaffold with small permeability. The permeability is too large, it is easy to wash out the cells and nutrients, which is also not conducive to tissue regeneration. The permeability was analyzed by computational fluid dynamics (CFD), the simulation process was completed in the software ANSYS 19.0 (ANSYS, America, https://www.pcsoft.com.cn/soft/194402.html). Considering that the analysis object was an incompressible fluid with constant density, the Navier–Stokes equation defined by Eq. (2) was used.

2 $$\left\{\begin{array}{c}\rho \frac{\partial{\varvec{\upsilon}}}{\partial t}+\rho \left({\varvec{\upsilon}}\bullet \nabla \right)\upsilon +\nabla P-\mu {\nabla }^{2}\upsilon =F\\ \nabla \bullet \upsilon =0\end{array}\right.$$

where ρ is the density of the fluid (kg/m^3^); v is the velocity of the fluid (m/s); t is the time (s); ∇ is the operator; P is the pressure (Pa); μ is the dynamic viscosity coefficient of the fluid (Pa s); F is the acting force (N).

**Figure 4:**
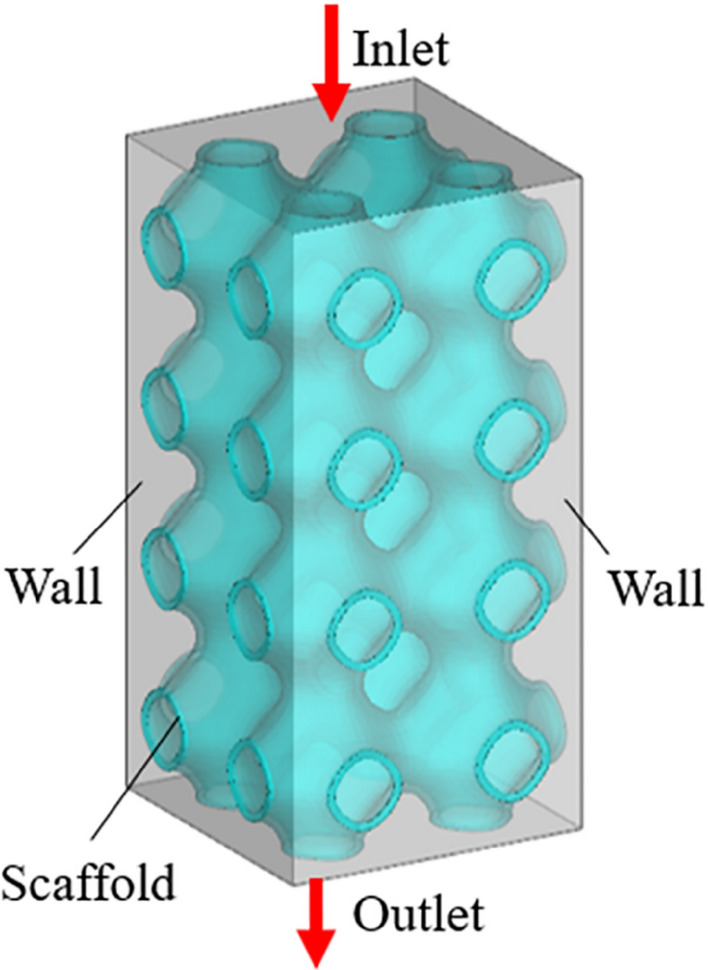
Boundary conditions of CFD analysis.

To simplify the simulation calculation and analysis, water was selected as the fluid domain material. At normal body temperature, the density and viscosity of water are 1000 kg/m^3^ and 1.45e−9 MPa s respectively^20,21^. The scaffolds were meshed using tetrahedral elements with a maximum mesh size of 0.002 mm. The boundary condition of the fluid model was shown in Fig. 4, the whole light color region was the fluid domain, and the green part was the scaffold model. The inlet velocity applied to the scaffold was set to 1 mm/s. Exit pressure is considered zero. The wall was assumed to have no slip^22^.

The fluid flow in the biomimetic bone scaffold was simulated by ANSYS18.2 (ANSYS, America), and the law of fluid flow in the scaffold was obtained. The pressure difference between the inlet and outlet of the scaffold and the permeability of the scaffold were calculated by Eqs. (3) and (4).

3 $$\Delta P={P}_{Inlet}-{P}_{outlet}$$

4 $$K=\frac{\mu \bullet \nu \bullet L}{\Delta P}$$

where K is the permeability coefficient (mm^2^); L is the characteristic length (mm); ΔP is the pressure difference (MPa).

### Cell sedimentation and adhesion

**Figure 5:**
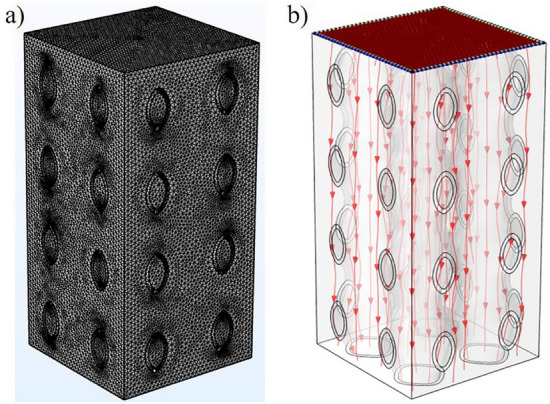
(**a**) Fluid domain meshing map; (**b**) cell initial distribution map.

The migration and settlement of cells in the scaffold can reflect the rationality of the scaffold design to a large extent. In this paper, the movement and adhesion of cells in the scaffold were simulated by Comsol software (Comsol, Sweden). The fluid model was divided by the tetrahedral mesh method, as shown in Fig. 5a). The upper surface uniformly meshed and the size was 0.05 mm. The cells were set to a spherical discrete phase with a diameter of 0.01 mm and a density of 1130 kg/m^3^^23^. The meshes on the upper surface were evenly distributed, as shown in Fig. 5b). It was assumed that the cell begins to settle from the upper surface under the action of gravity and drag, the initial velocity of the cell was 0 and the fluid velocity was 1 mm/s. The drag satisfies the Stokes equation defined by Eqs. (5) and (6).

5 $${F}_{D}=\frac{1}{{\tau }_{P}}\bullet m\bullet (u-\upsilon )$$

6 $${\tau }_{p}=\frac{\rho \bullet {d}^{2}}{18\mu }$$

where m is the particle mass (kg); d is the particle diameter (m); $\uprho$ is the particle density (kg/mm^3^); μ is the dynamic viscosity coefficient of the fluid (Pa s).

Adhesion occurs when the cell movement touches the side wall and the inner wall of the scaffold, and when it moves to the exit, it crosses the boundary. We used the counter of the software to count the number of adhesion in the process of cell migration to evaluate which group of scaffold structures is more conducive to cell adhesion.

### Statistical analysis

Analysis was performed using SPSS 20.0 software (SPSS Inc., Chicago, Il, USA). All the data were expressed as mean ± standard deviation and analyzed with the one-way ANOVA. In all cases, the results were considered statistically significant for p < 0.05.

## Results

### Compression test and simulation results

**Figure 6:**
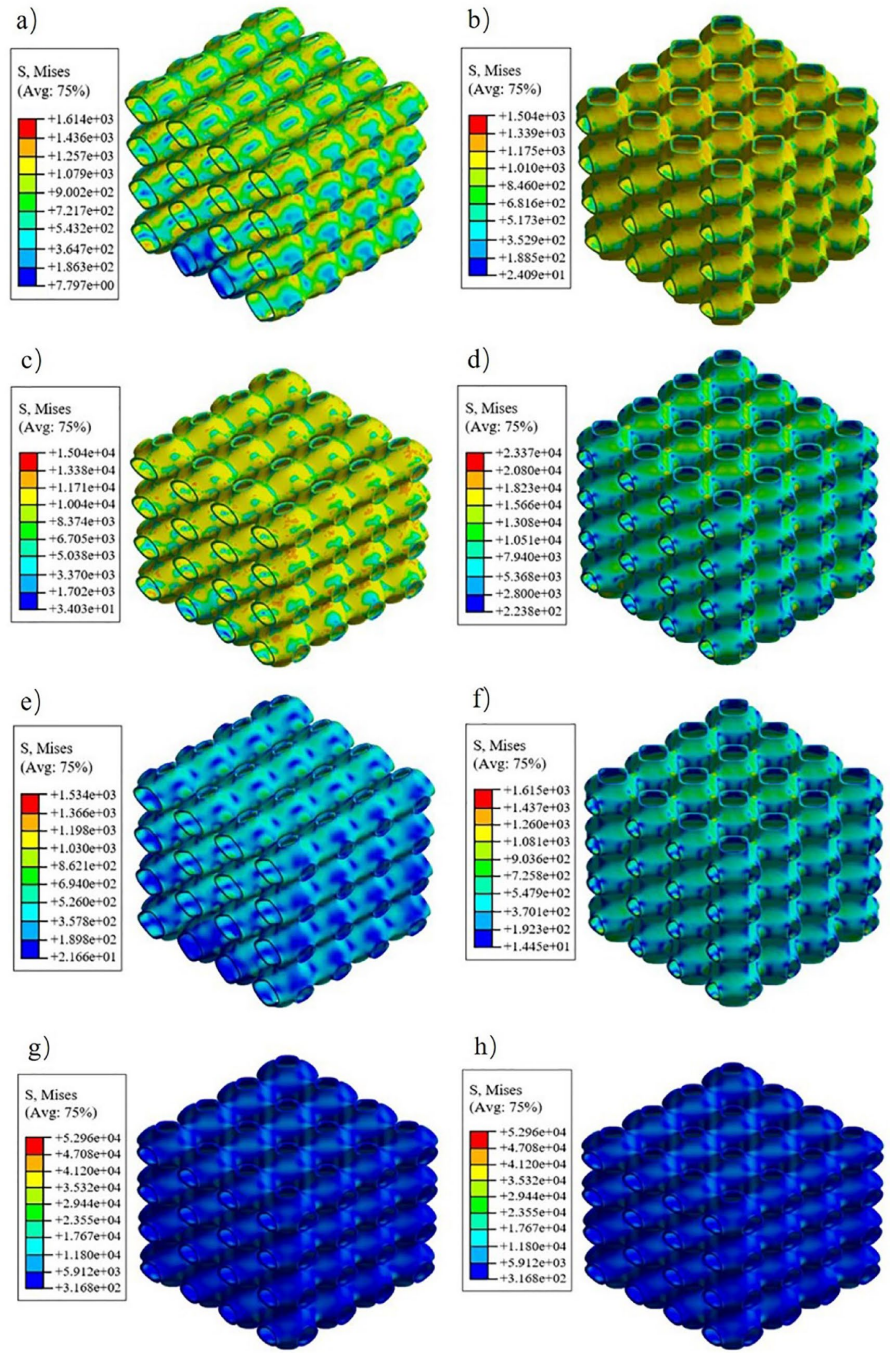
Finite element analysis cloud map: (**a**) the stress distribution of P1-3 scaffolds in Y direction; (**b**) the stress distribution of P1-3 scaffolds in Z direction; (**c**) the stress distribution of P1-2 scaffolds in Y direction; (**d**) the stress distribution of P1-2 scaffolds in Z direction; (**e**) the stress distribution of P2-3 scaffolds in Y direction; (**f**) the stress distribution of P2-3 scaffolds in Z direction; (**g**) the stress distribution of P1-1 scaffolds in Y direction; (**h**) the stress distribution of P1-1 scaffolds in Z direction.

**Figure 7:**
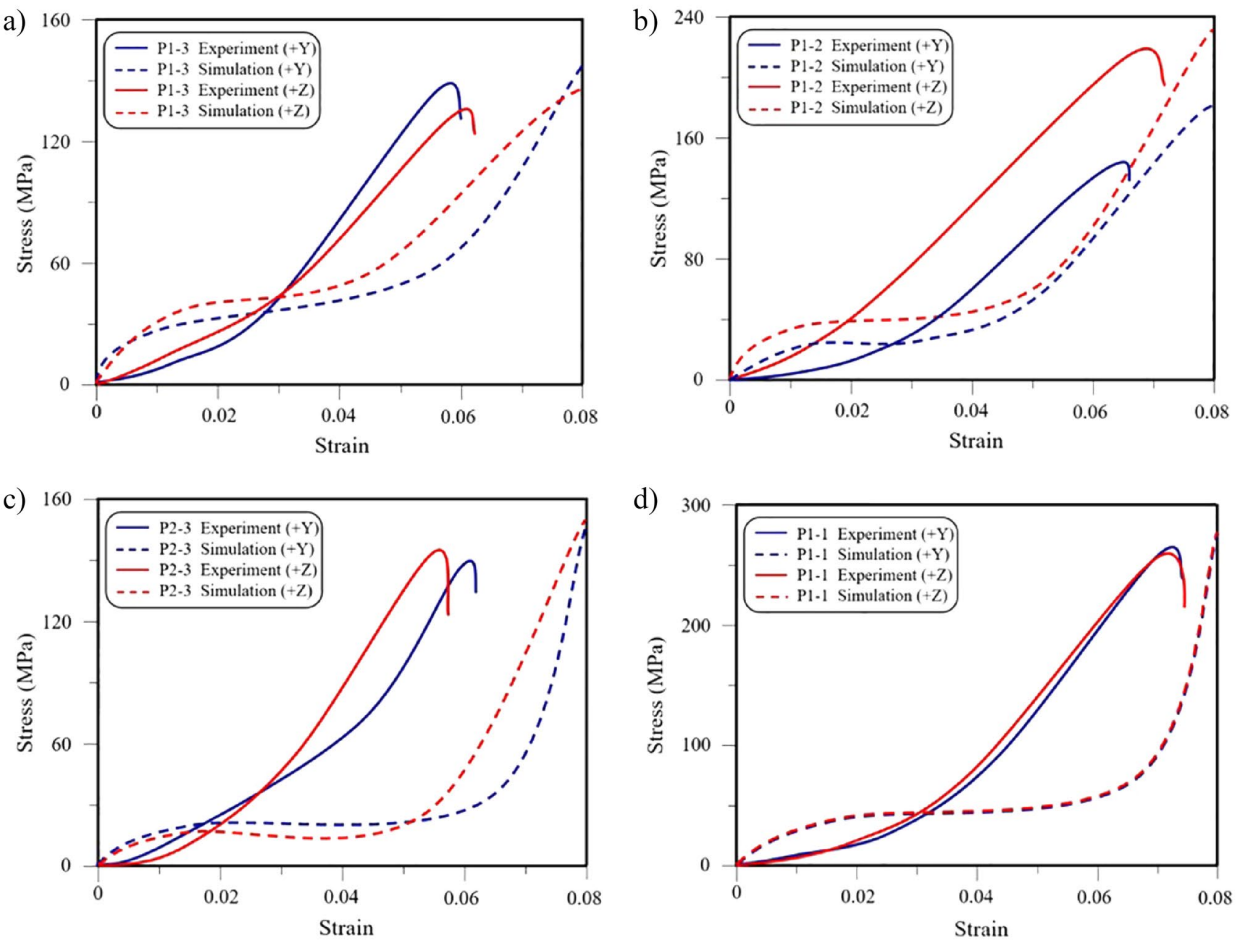
Stress–strain curve of scaffolds: (**a**) stress–strain curve by finite element simulation and compression test of P1-3; (**b**) stress–strain curve by finite element simulation and compress test of P1-2; (**c**) Stress–strain curve by finite element simulation and compress test of P2-3; (**d**) stress–strain curve by finite element simulation and compress test of P1-1.

The finite element compression simulation results were shown in Fig. 6. Figure 6a,c,e,f representing the stress distribution of P1-3, P1-2, P2-3, and P1-1 scaffolds in the Y direction respectively. Figure 6b,d,f,h represent the stress distribution of P1-3, P1-2, P2-3, and P1-1scaffolds in the Z direction respectively. It can be seen from Fig. 6 that the stress distribution range of the P1-3 scaffold in the Y direction is 7.797 to 1614 MPa and in the Z direction is 24.09 to 1504 MPa, and there is little difference between them. The range of stress distribution in the Y direction of the P1-2 scaffold is 34.03 to 15,040 MPa and the range of stress distribution in the Z direction is 223.8 to 23,370 MPa. There is a noticeable difference in stress distribution between them. The range of stress distribution in the Y direction of the P2-3 scaffold is 21.66 to 1534 MPa. The range of stress distribution in the Z direction is 14.45 to 1615 MPa, and there is no significant difference between them. The stress distribution range of the P1-1 scaffold in the Y direction and the Z direction is 316.8 to 5296 MPa, higher than the other three groups of scaffolds. To compare the data results more intuitively, the data obtained from numerical simulation and compression experiments were drawn into a stress–strain curve, as shown in Fig. 7. The values of Young's modulus and yield strength are shown in Table 2. From the results in the table, it can be found that the numerical simulation is different from the experimental results, but the overall change law is similar. In addition, an interesting phenomenon can be found that whether the aspect ratio is 1:3 or 2:3, there is little difference between their transverse and longitudinal mechanical properties. And the carrying capacity is the worst. When the shaft-diameter ratio is 1:2, the transverse and longitudinal mechanical properties are different, and the elastic modulus and yield strength are higher than those of the scaffold structure with a shaft-diameter ratio of 1:3 and 2:3. In addition, it can be seen that when the ratio of the shaft to diameter is not equal to 1:1, the elastic modulus and yield strength decreased significantly.

**Table 2:** Static characteristics of four groups of scaffolds (FE = finite element).

	P1-3 Y/Z	P1-2 Y/Z	P2-3 Y/Z	P1-1 Y/Z
Young’s modulus by FE simulation (GPa)	1.67/1.61	3.29/3.98	2.03/2.11	4.27/4.27
Young’s modulus by compressive testing (GPa)	1.8 ± 0.28/ 1.7 ± 0.25	2.4 ± 0.24/ 3.0 ± 0.17	1.8 ± 0.21/ 2.1 ± 0.18	4.5 ± 0.26/ 4.5 ± 0.24
Yield strength by FE simulation (MPa)	115.4/111.7	107.2/135.8	104.1/118.6	169.3/169.3
Yield strength by compressive testing (MPa)	107 ± 4.6/ 103 ± 5.1	94 ± 6.8/ 128 ± 6.5	99 ± 4.8/ 105 ± 5.4	152 ± 5.3/ 150 ± 6.2

### The results of permeability

**Figure 8:**
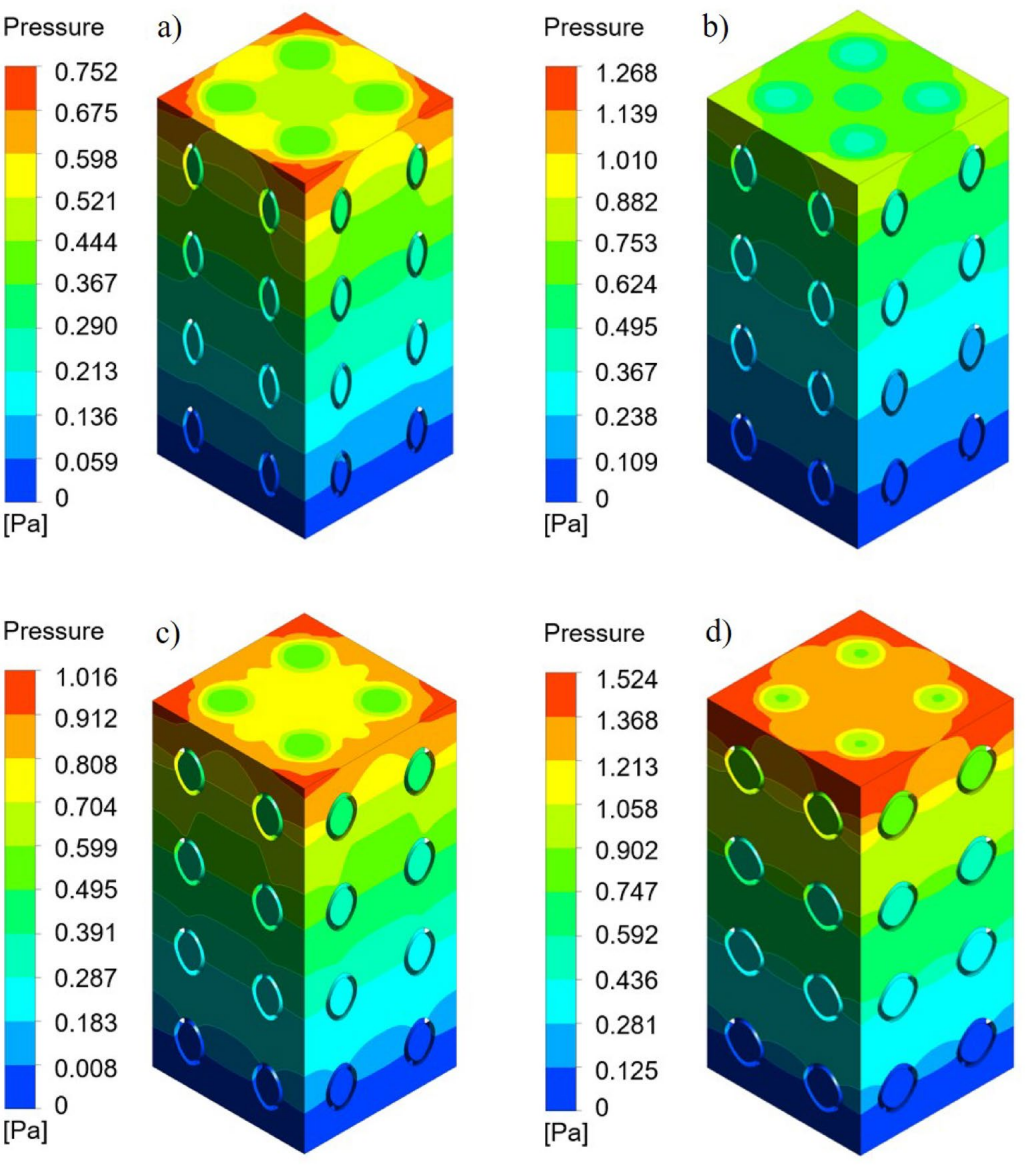
Four groups of scaffolds pressure drop cloud: (**a**) P1-3 pressure drop cloud; (**b**) P1-2 pressure drop cloud; (**c**) P2-3 pressure drop cloud; (**d**) P1-1 pressure drop cloud.

The pressure drop cloud picture of the four groups of scaffolds as shown in Fig. 8. Figure 8a–d is the pressure drop cloud maps of P1-3, P1-2, P2-3, and P1-1 scaffolds, respectively. It was observed that the cloud map distribution characteristics of the four groups of scaffold pressure distribution are similar, the highest pressure occurs at the entrance and gradually tends to zero in the exit area. From the simulation results, it can be observed that the pressure difference between the entrance and outlet of the P1-3 scaffold is the smallest, while the pressure drop of the P1-1 scaffold is the largest.

**Figure 9:**
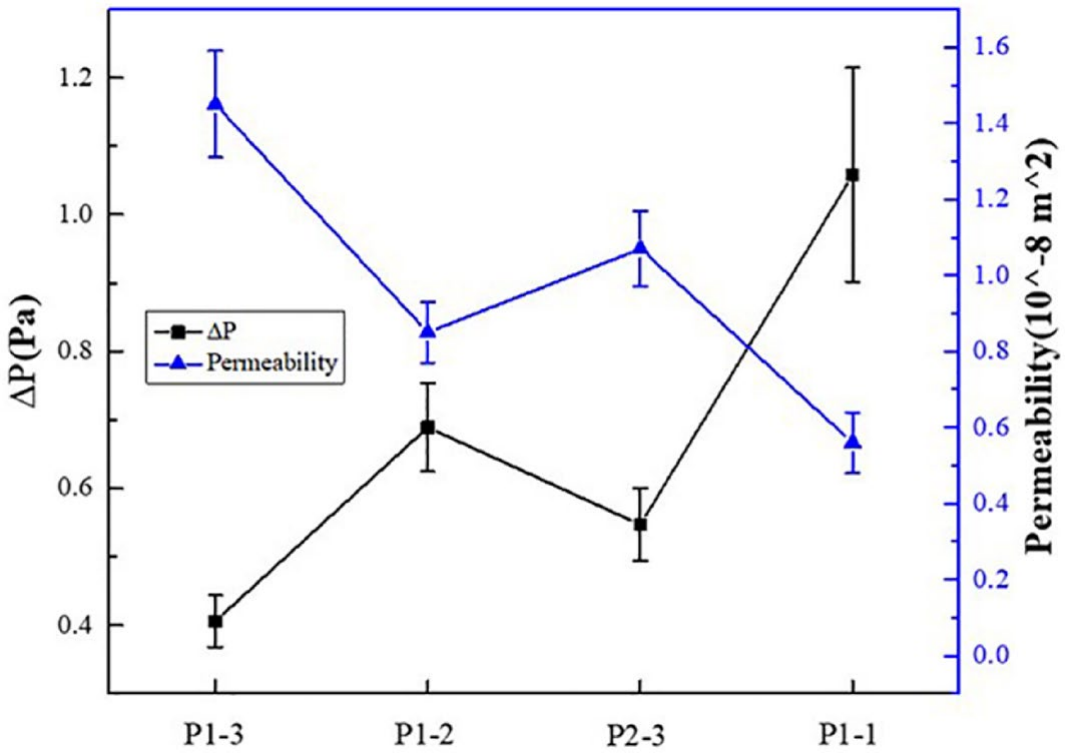
Pressure drop and permeability of four groups of scaffolds.

It can be seen from Eqs. (3) and (4) that under the same boundary conditions, the smaller the pressure drop is, the greater the permeability is, and the greater the pressure drop is, the smaller the permeability is. The pressure drop and permeability of each scaffold were calculated according to Eq. (3), and the results were shown in Fig. 9. The permeability of the P1-3 scaffold, P1-2 scaffold, P2-3 scaffold, P1-1 scaffold, and P1-1 scaffold was 1.28e−8 to 1.60e−8 m^2^, 0.72e−8 to 0.92e−8 m^2^, 0.94e−8 to1.18e−8 m^2^ and 0.46e−8 to 0.64e−8 m^2^, respectively.

### The results of cell sedimentation and adhesion

**Figure 10:**
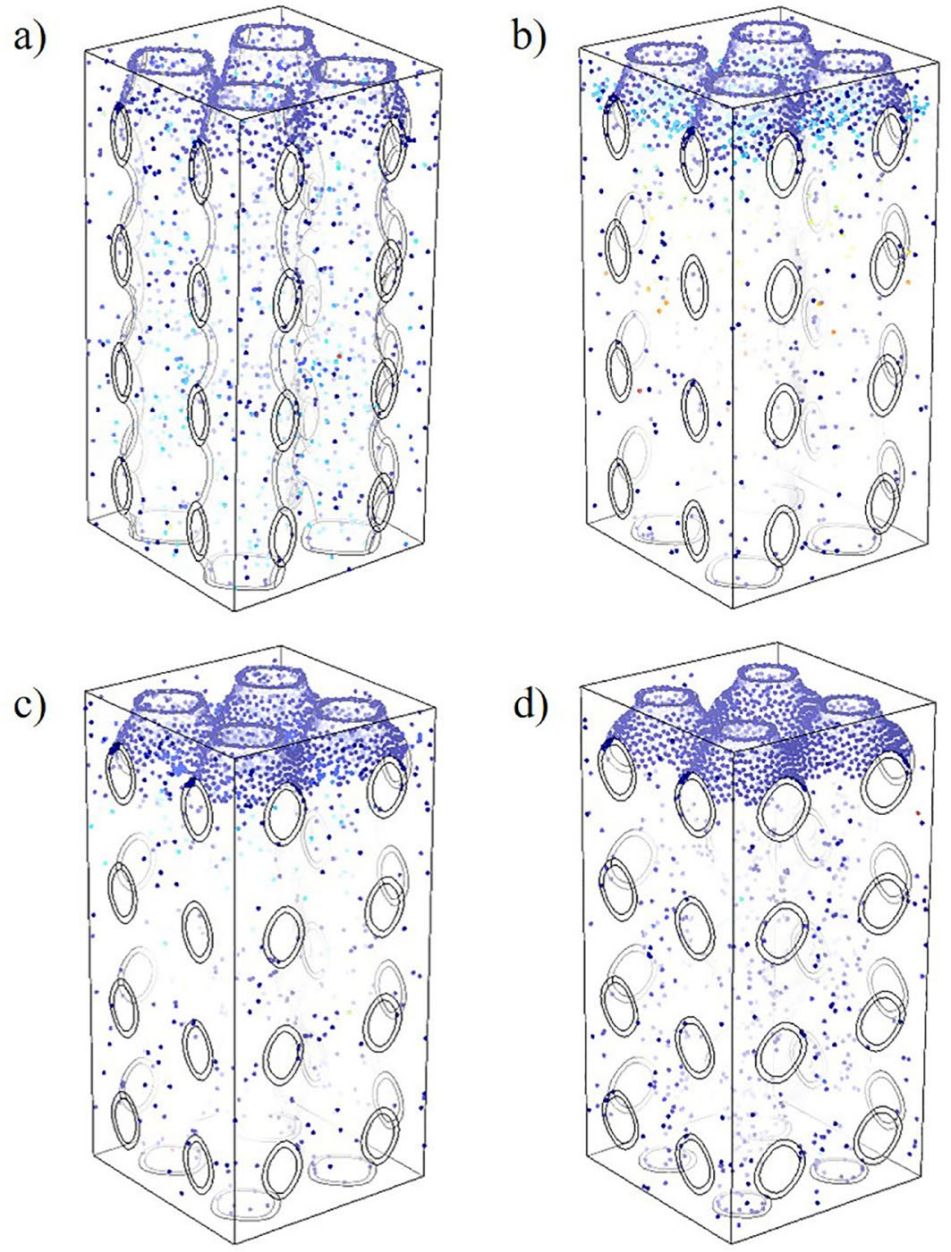
The cell sedimentation and adhesion diagrams: (**a**) cell sedimentation and adhesion maps of P1-3; (**b**) cell sedimentation and adhesion maps of P1-2; (**c**) cell sedimentation and adhesion maps of P2-3; (**d**) cell sedimentation and adhesion maps of P1-1.

**Figure 11:**
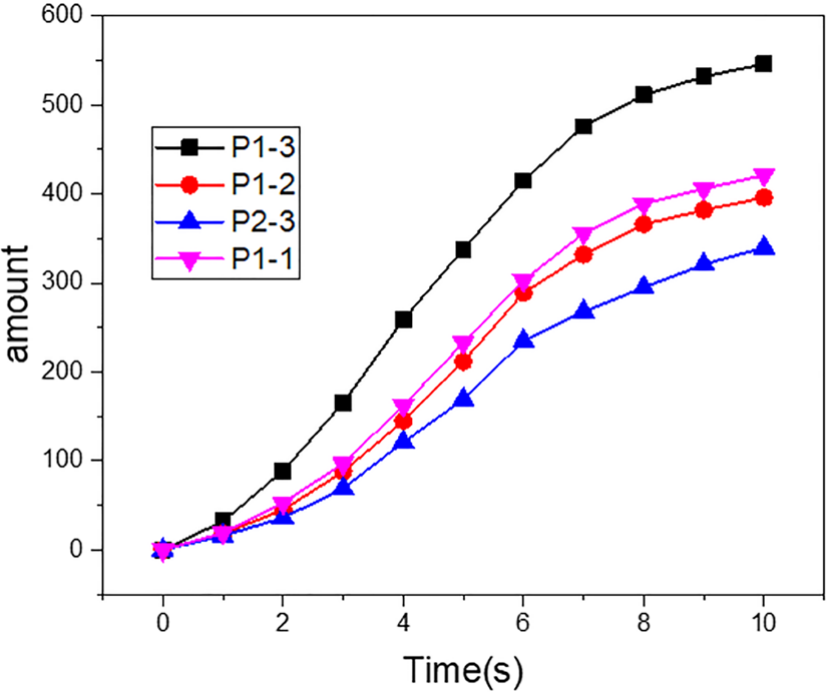
Statistics of cell adhesion of four groups of scaffolds.

The cells at the entrance of the four groups of scaffolds began to migrate under the action of fluid and gravity, crossing the exit boundary or adhering to the wall of the scaffold, as shown in Fig. 10. It can be observed from the results that P1-3 scaffold cells settled faster and more cells adhered to the inner wall in the same time period. The sedimentation rate of the P2-3 scaffold was relatively slow, and only a small number of cells adhered to the inner wall. To more intuitively compare the differences in cell sedimentation and adhesion among the four groups, the software counter was used to count the number of cell adhesion in the scaffold at the same time, and the results as given in Fig. 11. It can be seen that the amount of cell adhesion on the wall of P1-3 scaffold is the most at the same time, which is much higher than that of P2-3 scaffold. There was no significant difference in the number of cells adhered to the inner wall of P1-2 and P1-1 scaffolds.

## Discussion

For the designed bone scaffold, we expect that its performance parameters can match with real bone tissue of the human body as much as possible. Too low parameter values are not enough to meet the requirements, and too high parameter values will often bring adverse effects. The studies show that the elastic modulus of human trabecular bone is in the range of 0.1–4.5 GPa^24,25^, and the values of elastic modulus and yield strength are different between transverse and longitudinal, and the ratio of longitudinal elastic modulus to transverse elastic modulus is about 1.2–2^4,26,27^. The ratio of longitudinal yield strength to transverse yield strength is about 1.1–2.5^4,26,27^. According to the results of Table 2, it can be seen that the elastic modulus of P1-3, P1-2, and P2-3 scaffold is in this range. P1-1 scaffold elasticity modulus may be slightly higher. In addition, there was little difference between transverse and longitudinal elastic modulus and yield strength of P1-3 scaffold, which is close to 1:1, while the ratio of longitudinal to transverse elastic modulus and yield strength of P1-2 scaffold is about 1.25. The ratio of longitudinal to transverse yield strength of the P1-2 scaffold is about 1.36, which showed anisotropy, which is more consistent with the properties of human bone tissue.

The permeability of human cancellous bone is 0.5e−8 to 5e−8 m^2^^28–30^. According to the results of Fig. 9, the permeability of the P1-3 scaffold is 1.28e−8 to 1.60e−8 m^2^, and the permeability of the P1-2 scaffold is 0.72e−8 to 0.92e−8 m^2^, the permeability of P2-3 scaffold is 0.94e−8 to 1.18e−8 m^2^, the permeability of P1-1 scaffold is 0.46e−8 to 0.64e−8 m^2^. It can be found that the permeability of P1-3, P1-2, and P2-3 scaffolds meets the requirements, while the permeability of P1-1 scaffolds is slightly lower. It can be seen that the scaffold with a non-1:1 shaft-diameter ratio can enhance the ability of the scaffold to conduct fluid flow.

There is no literature on specific reference values for cell deposition and adhesion. However, the structural design of the scaffold should be more conducive to the migration and adhesion of cells, facilitate the proliferation and differentiation of cells inside the scaffold, and promote the repair of bone tissue in the defect site more quickly. Through the results of Figs. 10 and 11, it can be observed that the structural design of the P1-3 scaffold in the four groups is more conducive to the adhesion of cells to the inner wall of the scaffold, and the number of adhesion in the same time is significantly higher than that in the other three groups. On the other hand, the adhesion effect of P2-3 scaffolds on the inner wall of P2-3 scaffolds is the worst. Through our research, which can be found that the axis-diameter ratio of major and minor shafts is designed as a structure of non 1:1, its performance is quite different from that of the structure with a 1:1 axis-diameter ratio which is common in the study. The difference in mechanical properties is mainly due to the difference in stress distribution and stress concentration caused by different surface curvatures. The difference between fluid flow and cell migration is due to the difference in surface curvature, which results in different forces of fluid and cells in the scaffold, and leads to the difference in permeability and cell adhesion. Therefore, in the scaffold design, if researchers study the design of different axis-diameter ratios, regional density ratios, and regional porosity ratios, instead of focusing a lot of work on the simple, uniform, and isotropic structure, it may be more helpful to optimize the structure of biomimetic bone scaffold.

## Conclusion

In this study, four groups of scaffold structures with different ratios of the major axis to minor axis were designed. Through static stimulation, mechanical compression test, hydrodynamic simulation, and other methods, the performance characteristics of four groups of scaffolds were analyzed. Through research, it was found that the performance of the structure with a shaft-diameter ratio other than 1:1 is quite different from that of the common P1-1 structure with a shaft-diameter ratio of 1: 1. Compared with the P1-1 structure, the static properties of the other three structures are more suitable for human bone tissue. Among them, P1-2 scaffold with an axial diameter ratio of 1: 2, the longitudinal and transverse mechanical differences are the best match with human bone tissue. In addition, compared with the P1-1 scaffold, the permeability of the other three scaffolds is more consistent with human bone tissue. P1-3 scaffold structure is more conducive to cell adhesion on the inner wall of the scaffold. Undoubtedly, the research in this paper has a certain guiding significance for the optimal design of the scaffold structure. In the structural design of the scaffold, we can focus more on the curved surface structure with different shaft-diameter ratios to obtain better performance.

## Data Availability

All data generated or analysed during this study are included in this published article.
